# Excess Mortality Associated with Influenza among Tuberculosis Deaths in South Africa, 1999–2009

**DOI:** 10.1371/journal.pone.0129173

**Published:** 2015-06-15

**Authors:** Sibongile Walaza, Cheryl Cohen, Ananta Nanoo, Adam L. Cohen, Johanna McAnerney, Claire von Mollendorf, Jocelyn Moyes, Stefano Tempia

**Affiliations:** 1 National Institute for Communicable Diseases of the National Health Laboratory Service, Johannesburg, South Africa; 2 Influenza Division, Centers for Disease Control and Prevention, Atlanta, Georgia, United States of America; 3 Influenza Division, Centers for Disease Control and Prevention, Pretoria, South Africa; 4 Faculty of Health Sciences, University of the Witwatersrand, Johannesburg, South Africa; University of Cape Town, SOUTH AFRICA

## Abstract

**Background:**

Published data on the interaction between influenza and pulmonary tuberculosis (PTB) are limited. We aimed to estimate the influenza-associated mortality among individuals with PTB in South Africa from 1999–2009.

**Methods:**

We modelled the excess influenza-associated mortality by applying Poisson regression models to monthly PTB and non-tuberculosis respiratory deaths, using laboratory-confirmed influenza as a covariate.

**Results:**

PTB deaths increased each winter, coinciding with influenza virus circulation. Among individuals of any age, mean annual influenza-associated PTB mortality rate was 164/100,000 person-years (n = 439). The rate of non-tuberculosis respiratory deaths was 27/100,000 (n = 1125) for HIV-infected and 5/100,000 (n = 2367) for HIV-uninfected individuals of all ages. Among individuals aged <65 years, influenza-associated PTB mortality risk was elevated compared to influenza-associated non-tuberculosis respiratory deaths in HIV-infected (relative risk (RR): 5.2; 95% CI: 4.6–5.9) and HIV-uninfected individuals (RR: 61.0; CI: 41.4–91.0). Among individuals aged ≥65 years, influenza-associated PTB mortality risk was elevated compared to influenza-associated non-tuberculosis respiratory deaths in HIV-uninfected individuals (RR: 13.0; 95% CI: 12.0–14.0).

**Conclusion:**

We observed an increased risk of influenza-associated mortality in persons with PTB compared to non-tuberculosis respiratory deaths. If confirmed in other settings, our findings may support recommendations for active inclusion of patients with TB for influenza vaccination and empiric influenza anti-viral treatment of patients with TB during influenza epidemics.

## Introduction

In 2010, there were an estimated 8.8 million incident cases of tuberculosis globally [1]. Despite the availability of effective treatment, tuberculosis remains the second most common cause of infectious disease–related deaths worldwide, after human immunodeficiency virus (HIV) and acquired immune deficiency syndrome (AIDS) [2]. HIV is the most important risk factor for tuberculosis disease in South Africa. In South Africa, there were an estimated 390,000 incident cases of tuberculosis in 2011 of which 65% were co-infected with HIV [2].

Globally, it is estimated that annual influenza epidemics result in three to five million cases of severe illness, and 250,000–500,000 deaths [3]. In 2010 in South Africa, after tuberculosis, pneumonia and influenza was the second leading underlying natural cause of death [4]. HIV infection is an important risk factor for influenza–associated mortality. Individuals with AIDS experience substantially elevated (4–11 fold greater) influenza-associated mortality [5–7].

An increase in mortality associated with influenza virus circulation in countries with temperate climates including South Africa has been described for a number of outcomes including respiratory and cerebrovascular diseases and diabetes [6–13]. Review of data from the 1918 influenza pandemic suggests that many individuals who died had active tuberculosis [14, 15] and that underlying tuberculosis infection may have contributed to the elevated mortality observed in young adults [15, 16]. However, limited data are available on excess mortality associated with seasonal influenza infection among patients with tuberculosis. Assessing the mortality burden associated with influenza virus infection among tuberculosis-infected individuals may provide evidence to support prioritising tuberculosis patients for influenza vaccination and antiviral treatment.

We hypothesised that tuberculosis, a chronic lung infection, may cause lung damage leading to reduced lung capacity and thus impairing the ability to cope with viral infections such as influenza and potentially increasing the severity of illness associated with influenza virus infection. Since influenza virus infections are rarely laboratory-confirmed and influenza-related deaths may be attributed to other comorbid conditions or secondary complications, we applied modelling approaches as previously described [6, 7] to estimate the influenza-associated mortality among individuals who died with pulmonary tuberculosis (PTB) in South Africa from 1999 through 2009.

## Methods

### Selection of the study populations

To investigate if there was an increased risk of mortality associated with influenza virus infection in patients with tuberculosis we estimated the rate of influenza-associated mortality among PTB deaths and compared it to the rate of influenza-associated mortality among non-tuberculosis respiratory deaths. In South Africa from prospective hospital-based surveillance studies conducted in 2009–2013 >80% of TB-associated deaths were in individuals coinfected with HIV (Personal communication Cohen 2014), and HIV was a risk factor for influenza-associated mortality [5–7]. To investigate whether the rate of influenza-associated mortality among PTB deaths was greater than expected simply as a result of underlying HIV infection, we compared the rate of influenza-associated mortality among PTB deaths to the rate of influenza-associated mortality among non-tuberculosis respiratory deaths in HIV-infected and-uninfected individuals. In addition, we estimated the rates of influenza-associated mortality among PTB and non-tuberculosis respiratory deaths in individuals aged <65 and ≥65 years. In individuals aged ≥65 years we expected a minimal effect of HIV infection on influenza-associated mortality because of the relatively low population HIV prevalence in this age group (0.6% ≥65 years vs. 11% in <65 years in 2009) [17]. This is also reflected amongst patients with laboratory-confirmed PTB who died identified through prospective surveillance from 2009–2013 (HIV prevalence 29% (2/7) in patients ≥65 years who died vs. 85% (73/86) in patients <65 years who died) (Personal Communication, Cohen 2014).

### Mortality data and population denominators

We obtained mortality data coded according to the *International Classification of Diseases*, *Tenth Revision* (ICD-10) from Statistics South Africa for 1999 through 2009 [4]. We compiled age-specific (<65 and ≥65 years of age) monthly mortality time series data. For each individual, we evaluated multiple underlying and contributing causes of death. Patients in whom the codes for PTB (ICD-10: A15-A16 and P37) appeared anywhere as an underlying or contributing cause of death were classified as PTB. Patients in whom the codes for PTB did not appear as an underlying or contributing cause of death but an underlying or contributing cause of death was a respiratory illness (ICD-10: J00-J99) were classified as non-tuberculosis respiratory deaths. To account for a systematic misclassification of cause of death that occurred between 1999 and 2005 in children aged 1–11 months we reallocated post-neonatal causes of death (i.e. ICD-10: P23—congenital pneumonia) to more appropriate causes of infant death (i.e. ICD-10: J18 –pneumonia, organism unspecified) as recommended by Statistics South Africa [18]. In addition, we adjusted for underreporting of deaths in children <5 years of age from 1999 to 2006 using the year-specific estimates of proportion of underreported deaths provided by Darikwa and Dorrington [19]. According to these estimates, data completeness increased from 61% in 1999 to 89% in 2006. From 2007 underreporting was estimated to be less than 5% [20].

Population denominators were obtained from Statistics South Africa [21] while age- and year-specific data on HIV prevalence in the population and highly active antiretroviral treatment (HAART) coverage among HIV-infected individuals were obtained from the Actuarial Society of South Africa (ASSA) AIDS and Demographic model [17].

We obtained the annual number of tuberculosis cases from the National Strategic Plan for HIV, STI and TB: 2012 to 2016 [22]. Because these data were not available by age group, we estimated the proportion of tuberculosis cases aged <65 and ≥65 years based on national data on age-distribution of laboratory-confirmed tuberculosis cases from the corporate data warehouse of the National Health Laboratory Services (NHLS-CDW). The number of tuberculosis-uninfected individuals in the population was obtained by subtracting the estimated number of tuberculosis cases from the number of individuals in the population within each age group.

### Influenza surveillance data

We obtained influenza virus data including types and subtypes from influenza-like-illness surveillance [23]. In addition, we acquired data on influenza testing from a national database, the NHLS-CDW, which included all patients tested for respiratory viruses in the public sector in South Africa [6]. We considered an influenza type or subtype to be dominant during the influenza season when it accounted for >50% of the circulating viruses.

### Estimation of influenza-associated mortality

To estimate the influenza associated mortality, we fitted age-specific generalized regression models (GLM) to the number of monthly deaths using a Poisson distribution and an identity link as previously described [6, 7]. The identity link was selected because it is considered the most biologically plausible link to model the impact of pathogen circulation on mortality [24–27]. The full model (Model 1) included covariates for time trends and seasonal variation as well as viral circulation as follows:

$$\begin{array}{l} E\left(Y_{i}{}_{,t}\right)=\beta_{0,i}+\beta_{1,i}\left[t\right]+\beta_{2,i}\left[t^{2}\right]+\beta_{3,i}\left[t^{3}\right]+\beta_{4,i}\left[t^{4}\right]+\beta_{5,i}\left[\text{sin}\left(2t\pi /12\right)\right]+\beta_{6,i}\left[\text{cos}\left(2t\pi /12\right)\right]\\ +\beta_{7,i}\left[Influenza(t)\right]+\varepsilon_{i}(t) \end{array}$$ (1)

Where *E(Y*_*i*,*t*_ *)* represents age-specific number of deaths in age group *i* and month *t*; *β*_*0*,*i*_ is the age-specific model constant; *β*_*1*,*i*_ to *β*_*4*,*i*_ are age-specific coefficients associated with time trends (linear to quartic polynomial terms); *β*_*5*,*i*_ and *β*_*6*,*i*_ are age-specific coefficients associated with harmonic terms accounting for annual background seasonal variations; *β*_*7*,*i*_ is the age-specific coefficients representing the contribution of influenza virus to mortality and *ε*_*i*_ *(t)* is the age-specific error term. *Influenza(t)* is a proxy for monthly viral activity, estimated as the monthly number of specimen testing positive for influenza over the annual number of specimens tested. We used standardization by the annual total of all specimens tested, to reduce possible bias associated with differences in specimen sampling and laboratory methods over time [28]. Model selection procedures included the assessment of model fit considering the inclusion of polynomial (1^st^ to 6^th^ degree) and harmonic terms. The final model (Model 1) was that for which the Akaike value was minimized, that is, the model that provided best fit to the data whilst maintaining parsimony. We also considered b-spline instead of polynomial terms but polynomial terms provided the best fit to the South African data.

To estimate the excess mortality associated with influenza, we subtracted predicted monthly deaths from a full model incorporating the influenza detection rate from an expected baseline. The baseline was obtained by setting the influenza covariate to 0 and the annual influenza excess mortality was obtained as the sum of the monthly excess mortality estimates for each year. We obtained the 95% confidence interval (CI) for the estimated excess mortality using bootstrap resampling on blocks of calendar years (12 months block resampling with replacement) over 1000 replications [6, 7, 27]. For each resampled dataset we refitted the Poisson regression model and the 95% CI were obtained from the 2.5^th^ and 97.5^th^ percentiles of the estimated influenza-associated mortality from the 1000 resampled datasets.

In South Africa, the diagnosis of HIV/AIDS is rarely coded on the death certificate [18]. To assess changes in annual influenza excess mortality rates among non-tuberculosis respiratory deaths (as obtained from Model 1) in relation to the HIV prevalence in the population, we fitted separate multivariable GLM (Model 2) for annual non-tuberculosis respiratory influenza-associated mortality rates by age group as previously described [6, 7] The following model was used:

$$E\left(Y_{i}{}_{,t}\right)=\alpha_{i}\left(\begin{array}{l} \beta_{0,i}+\beta_{1,i}[t]+\beta_{2,i}[t^{2}]+\beta_{3,i}[Influenza\_Subtype\;(t)]\\ +\beta_{4,i}[HIV_{i}(t)]+\beta_{5,i}[HAART_{i}(t)]+\varepsilon_{i}(t) \end{array}\right)$$ (2)

Where *E(Y*_*i*,*t*_ *)* represents the age-specific number of influenza-associated excess deaths in age group *i* and year *t* (as obtained from Model 1); *α*_*i*_ is an offset representing the population size of age group *i*; *β*_*0*,*i*_ is the age-specific intercept; *β*_*1*,*i*_ and *β*_*2*,*i*_ are age-specific coefficients associated with time trends (linear and quadratic) included to account for potential variations of health indicators unrelated to influenza, HIV prevalence or HAART coverage in the population; *β*_*3*,*i*_ is the age-specific coefficient associated with dominant seasonal influenza type/subtype each year (categorical variable with A(H3N2)-dominant years as reference group vs. A(H1N1) or B); *β*_*4*,*i*_ is the coefficient associated with the HIV prevalence in the population in age group *i* and year *t*; *β*_*5*,*i*_ is the coefficient associated with HAART coverage among HIV-infected individuals in the population in age group *i* and year *t*; and *ε*_*i*_ *(t)* is the age-specific error term. We estimated the excess mortality associated with HIV-infection among influenza-associated deaths by subtracting an expected baseline from the Model 2 annual estimates. The baseline was obtained by setting the HIV and HAART covariates to 0.

Mortality rates in individuals with tuberculosis were obtained by dividing the estimated influenza-associated deaths by the number of individuals with tuberculosis within each age group. Mortality rates for non-tuberculosis respiratory deaths by HIV status were obtained by dividing the estimated excess deaths by the population at risk (i.e. population without tuberculosis) within each age-group and HIV-status category. Age-specific relative risks for influenza-associated mortality among individuals with PTB compared to influenza-associated non-tuberculosis respiratory mortality among HIV-infected and-uninfected individuals were estimated using log-binomial regression. The statistical analysis was implemented using STATA version 12 (StataCorp, Texas, USA).

### Ethics

Since this study used only publicly-available mortality data and de-identified and aggregated laboratory data, this analysis was considered to be exempt from human subjects’ ethics review.

## Results

### Mortality data

From 1999 through 2009, a mean of 550,769 (range 418,729–621,002) deaths occurred annually among South African individuals. Of these deaths, 118,995 (22%) were non-tuberculosis respiratory, and 63,596 (12%) were PTB (Table 1). Among individuals aged <65 years the mean annual mortality rate per 100,000 person-years was 205 for non-tuberculosis respiratory deaths and 23,154 for PTB deaths. In individuals aged ≥65 years the mean mortality rate per 100,000 person-years was 1202 for non-tuberculosis respiratory deaths, and 55,479 for PTB deaths (Table 1).

**Table 1:** Mean annual non-tuberculosis respiratory and pulmonary tuberculosis deaths in South Africa, 1999–2009.

Cause of death	Deaths	
	Number Mean (Range)	Rate^1^ Mean (Range)
**Non-tuberculosis respiratory**		
<65	92,416 (63,365–108,032)	205 (149–239)
≥65	26,579 (23,047–29,756)	1202 (1148–1264)
All Age	118,995 (86,412–134,549)	252 (195–282)
**Pulmonary tuberculosis**		
<65	59,387 (33,445–71,754)	23,154 (15,606–28,178)
≥65	4208 (3706–4986)	55,479 (40,927–86,402)
All Age	63,596 (37,151–76,269)	24,124(16,365–29,925)

^1^Death rates due to non-tuberculosis respiratory disease among individuals without tuberculosis per 100,000 person-years and death rate due to pulmonary tuberculosis among individuals with pulmonary tuberculosis per 100,000 person-years

**Fig 1:**
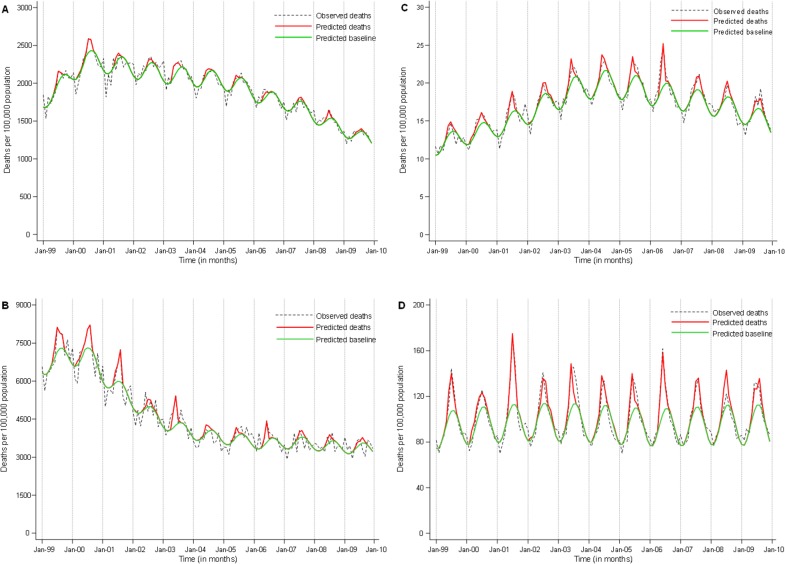
Monthly observed deaths, predicted deaths and predicted baseline (Poisson model), South Africa, 1999–2009. **A:** Pulmonary tuberculosis deaths in individuals <65 years of age. **B:** Pulmonary tuberculosis deaths in individuals ≥65 years of age; **C** Non-tuberculosis respiratory deaths in individuals <65 years of age. **D** Non-tuberculosis respiratory deaths in individuals ≥65 years of age.

**Fig 2:**
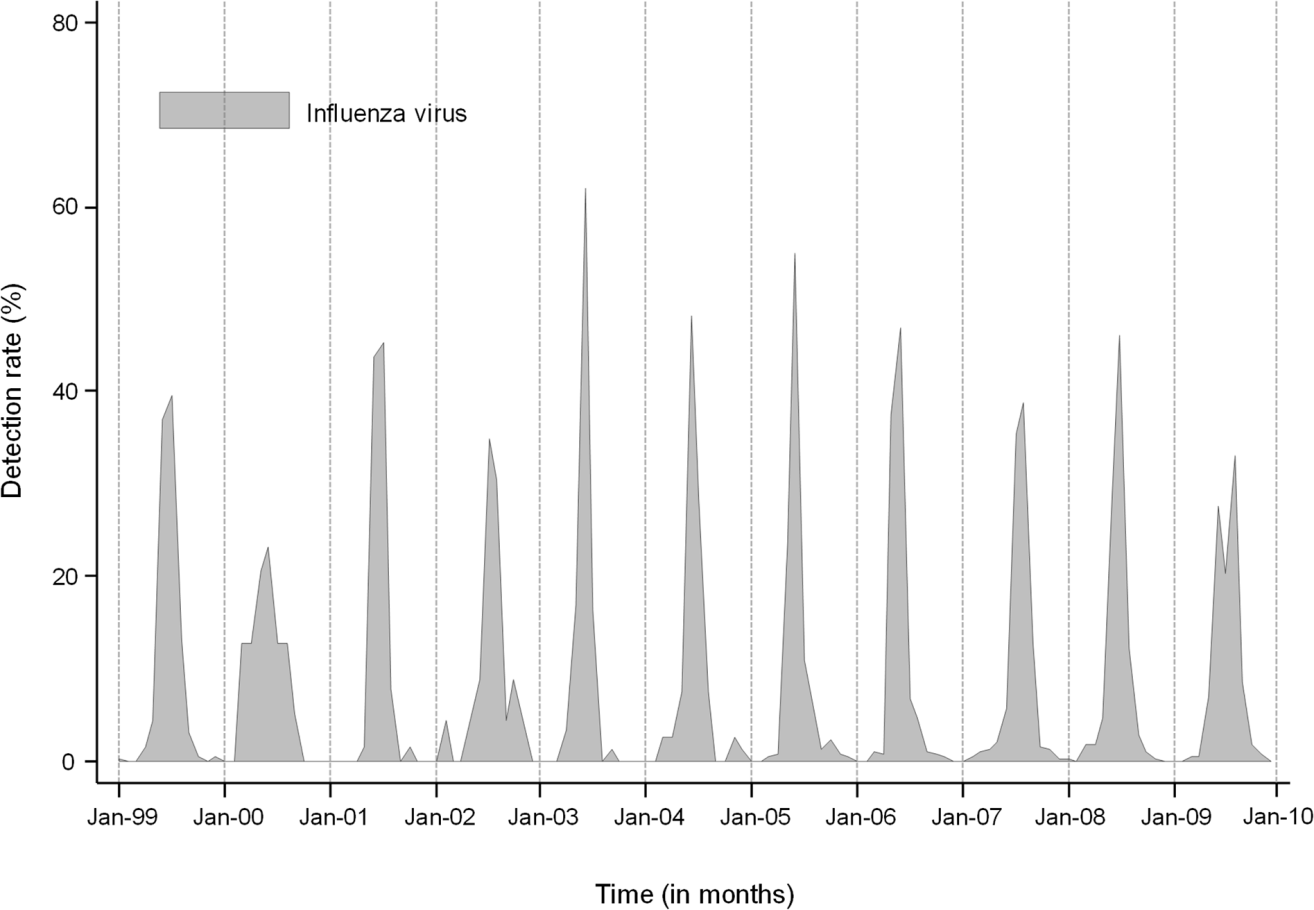
Monthly detection rate (i.e., monthly number of positive specimens divided by total specimens) of influenza (all ages) in South Africa, 1999–2009.

PTB deaths were seasonal and increased each winter, coinciding with the period of influenza virus circulation (Figs 1A, 1B and 2). There was a decreasing trend in PTB mortality rates over the study period for individuals aged <65 and ≥65 years (Fig 1A and 1B). Non-tuberculosis respiratory deaths were also highly seasonal. Among individuals aged <65 years non-tuberculosis respiratory deaths increased until 2006 following the increasing HIV prevalence in the population and then began to decrease following the progressive roll out of HAART (Fig 1C). In the elderly, an age group where the HIV prevalence is lowest, the rates were stable over time (Fig 1D).

### Influenza and laboratory surveillance

A mean of 3337 (range 227–15,321) samples were tested for influenza viruses annually and testing increased over time. The mean annual number of specimens testing positive for influenza viruses was 988 (30%). Over the study period, the influenza season peaked between May and August with 9 of the 11 years experiencing peak activity in June-July (Fig 2).

### Influenza-associated mortality

Among individuals <65 years of age the mean annual influenza-associated mortality rates per 100,000 person-years were 141 (n = 375) among PTB deaths, 27 (n = 1125) among HIV-infected non-tuberculosis respiratory deaths, and 2 (n = 937) among HIV-uninfected non-tuberculosis respiratory deaths (Table 2). In this age group, the risk of influenza-associated mortality was greater among PTB deaths compared to HIV-infected non-tuberculosis respiratory deaths (relative risk (RR): 5.2; 95% CI: 4.6–5.9) and HIV-uninfected non-tuberculosis respiratory deaths (RR: 61.0; CI: 41.4–91.0).

**Table 2:** Mean annual influenza-associated excess mortality among pulmonary tuberculosis and non-tuberculosis respiratory deaths in South Africa, 1999–2009.

Cause of death	Estimated mean annual influenza-associated deaths		
	Total		
	Number Mean (95% CI)	Rate per 100,000 person years Mean (95% CI)	Percentage mortality over model baseline Mean (95% CI)
**Pulmonary tuberculosis** ^**1**^			
<65	375 (213–531)	141 (122–150)	1.8 (1.6–2.0)
≥65	64 (35–86)	836 (555–984)	4.4 (3.4–5.6)
All Age groups	439 (248–617)	164 (144–174)	2.0 (1.8–2.2)
**Non-tuberculosis respiratory** ^**2**^ ^.^			
<65	2063 (1455–2799)	4.6 (4.4–4.8)	6.3 (6.1–6.6)
≥65	1430 (1149–1733)	64 (61–68)	14.9 (14.2–15.6)
All age groups	3493 (2604–4532)	7.4 (7.2–7.6)	8.3 (8.0–8.6)
**Non-tuberculosis respiratory** ^**2**^ **HIV+**			
<65	1125 (794–1525)	27 (26–29)	N/A
≥65	Not determined	Not determined	N/A
All age groups	1125 (794–1525)	27 (26–29)	N/A
**Non-tuberculosis respiratory** ^**2**^ **HIV-**			
<65	937 (661–1273)	2 (1.6–3.1)	N/A
≥65	1430 (1149–1733)	64 (61–68)	N/A
All age groups	2367 (1810–3006)	5 (3.1–5.3)	N/A

^1^Any individual with PTB

^2^Any individual without PTB

Among individuals ≥65 years of age the mean annual influenza-associated mortality rates per 100,000 person-years were 836 (n = 64) among PTB deaths and 64 (n = 1430) among HIV-uninfected non-tuberculosis respiratory deaths (RR: 13.0; 95% CI: 12.0–14.0). In this age group, where the HIV burden is lowest, our model (Model 2) did not estimate non-tuberculosis respiratory influenza-associated mortality among HIV-infected individuals.

## Discussion

In South Africa, PTB deaths were seasonal, with higher mortality rates observed during the winter months, coinciding with the period of influenza virus circulation. Among individuals aged <65 years, the estimated mortality rate associated with influenza virus infection was greater among PTB deaths compared to non-tuberculosis respiratory deaths in HIV-infected and–uninfected individuals suggesting that the increased risk of death among PTB deaths was not only related to underlying HIV-infection. The estimated mortality rate associated with influenza virus infection was greater among PTB deaths compared to non-tuberculosis respiratory deaths also among individuals aged ≥65 years; an age group where the population HIV prevalence is low. These findings suggest that PTB may be an independent risk factor for increased influenza-associated mortality.

The findings of increased risk of influenza-associated mortality in patients with PTB are consistent with ecological data prior to the HIV era, where elevated influenza-related mortality was reported among tuberculosis-infected patients during the 1918 influenza pandemic [15, 16, 29]. A harvesting effect was described by Noymer [14] using historical data from the United States, demonstrating that many of those who died during the 1918 influenza pandemic had underlying tuberculosis. This led to decreased TB mortality and transmission in the years that followed the influenza pandemic.

Mice models have shown that co-infection with influenza and Bacillus Calmette–Guérin in the lung led to exacerbation of the pulmonary disease and that pulmonary co-infection with influenza reduces production of protective T cell responses against intracellular mycobacterium [30]. A case series of patients who died during the 2009 influenza pandemic in South Africa described underlying tuberculosis in 10% of deaths which was greater than the population prevalence of tuberculosis [31]. Another study from South Africa showed that patients co-infected with tuberculosis and influenza as compared to patients with tuberculosis only were at increased risk of death (adjusted relative risk ratio (aRRR) 3.2 95% CI 1.1–10.0). This risk was higher in individuals with a more chronic presentation (aRRR 5.48, 95% CI 1.2–25.4) [32]. However, a study from Thailand, which included only patients with an acute presentation, did not identify any increased risk of severe outcomes or mortality in patients co-infected with influenza and tuberculosis [33]. These findings suggest that the mechanism by which tuberculosis may predispose to increased risk of influenza-associated mortality may be associated with underlying lung damage caused by tuberculosis in individuals with PTB rather than a systemic or general immunologic effect.

In South Africa individuals with PTB have a high prevalence of HIV co-infection [2] and HIV infection is a known risk factor for increased influenza-associated mortality [5]. In our study we found an increased risk of influenza-associated mortality among PTB compared to non-tuberculosis respiratory deaths irrespective of HIV status. Nonetheless, it is possible that individuals with PTB may be at a more advanced stage of HIV infection compared to HIV-infected non-tuberculosis respiratory cases. This may make PTB cases more at risk of influenza-associated mortality because of lower CD4+ T cell counts and not necessarily because of PTB. However, Sonnenberg, et al. reported that the TB incidence doubled within the first year of HIV infection, suggesting that TB is a relatively early disease among HIV-infected individuals [34]. In addition an increased risk of influenza-associated mortality among PTB compared with non-tuberculosis respiratory deaths was found even among elderly individuals ≥65 years of age where the HIV prevalence is low [17].

Marked secular trends were observed in PTB deaths in individuals of all ages as well as non-tuberculosis respiratory deaths in individuals aged <65 years. These are likely a result of several contributing factors including more widespread availability of highly active antiretroviral therapy since 2004 [35], and strengthened services for the diagnosis and management of tuberculosis since 2003 [22].

Our study is subject to limitations that warrant discussion. First, because of poor recording of HIV infection in the death register in South Africa [36], we utilized indirect methods to assess the mortality burden associated with influenza among HIV-infected and-uninfected non-tuberculosis respiratory deaths. While the HIV epidemic in South Africa is considered to be a major factor responsible for the increased mortality rates observed over the years, the lack of time series data for other potential comorbidities/risk factors may have resulted in overestimating the increased risk of death associated with HIV infection. Nonetheless, in a prospective hospital-based severe acute respiratory illness surveillance study from 2009–2013 the HIV prevalence among laboratory-confirmed influenza positive and tuberculosis negative deaths was 59% (10/17) and 0% (0/5) among individuals <65 and ≥65 years of age, respectively (Cohen, personal communication, July 2014). This prevalence is similar to the estimated HIV prevalence in our study from Model 2: 55% (1125/2063) and 0% (0/1430) among influenza-associated non-tuberculosis respiratory deaths in the <65 and ≥65 year age groups, respectively. Second, while we utilized methods suggested by Statistics South Africa to account for the systematic misclassification of cause of death in infants from 1999–2005 and deaths underreporting from 1999–2006, such adjustment may have introduced potential biases in the early years of our study. Third, we did not have individual level data and were not able to further explore whether the PTB deaths which we observed were in individuals with chronic underlying tuberculosis who experienced an acute exacerbation as a result of superimposed influenza infection. For PTB and non-tuberculosis respiratory deaths we relied on the diagnosis recorded on the death certificates with possible misclassification of cause of death. However, this is unlikely to be sufficient to account for the observed increases. Previous studies from South Africa have shown that there is no winter increase in mortality for “control” diagnoses such as cancer for which no association with influenza has been demonstrated [12]. Fourth, our modelling approach assumes that all excess deaths occurring above the model baseline are attributable to influenza. Influenza-associated mortality could in part be associated with other infections such as *Streptococcus pneumoniae* that have been shown to interact with influenza leading to severe outcomes [37]. However, this effect would be expected to occur among PTB and non-tuberculosis respiratory cases alike. Fifth, we obtained denominators for the number of individuals with PTB from national published estimates [22]. Some cases of PTB may not have been recorded on the national register and this could have led to our denominators of numbers of individuals with PTB being too low which would have led to an overestimation of rates of death in individuals with tuberculosis. However similar estimates of tuberculosis case numbers in the South African population have been found using estimates extrapolated from laboratory-confirmed cases [38]. Sixth, we were not powered to estimate excess influenza mortality in more refined age groups, owing to the low prevalence (and associated mortality) of tuberculosis in individuals <15 years. Lastly, among PTB deaths, we could not assess the influenza-associated mortality by HIV status because in our setting the vast majority of tuberculosis patients are positive for HIV and we could not disaggregate the effect of HAART and improved TB detection and treatment in this group.

In conclusion, we reported an increased risk of influenza-associated mortality among individuals with PTB. If confirmed in other countries and settings, these findings may support recommendations for active inclusion of patients with PTB for influenza vaccination (in addition to existing recommendations for vaccination of HIV-infected individuals [39]) and empiric anti-viral treatment for influenza among patients with PTB presenting with acute respiratory symptoms during influenza epidemics. Nonetheless, there are limited published data on the effectiveness or efficacy of influenza vaccination in patients with tuberculosis, according to our knowledge none among those co-infected with HIV. Studies of vaccine efficacy in this group of individuals should be implemented.
